# Impact of psychotropic *pro re nata* prescription-monitoring programme on prescriptions for inpatients with psychiatric disorders: a retrospective observational study

**DOI:** 10.1186/s12888-025-06508-w

**Published:** 2025-01-17

**Authors:** Yoshitaka Saito, Kyohei Sumida, Hiroyuki Muraoka, Satoru Oishi, Ryutaro Suzuki, Taiyo Nishikawa, Shin Miyake, Yukihiro Tanno, Yuki Tobita, Katsuya Otori, Ken Inada

**Affiliations:** 1 Department of Psychiatry, Kitasato University, School of Medicine, 1-15-1 Kitasato, Minami-Ku, Sagamihara, Kanagawa 252-0374 Japan; 2 Department of Pharmacy, Kitasato University Hospital, 1-15-1 Kitasato, Minami-Ku, Sagamihara, Kanagawa 252-0374 Japan; 3 Department of Psychiatry, Kitasato University Graduate School of Medical Sciences, 1-15-1, Kitasato, Minami-Ku, Sagamihara-Shi, Kanagawa, 252-0374 Japan; 4 Department of Psychiatry, Division of Integrated Psychosocial Care in Community and Child Psychiatry, Kitasato University, School of Medicine, 1-15-1 Kitasato, Minami-Ku, Sagamihara, Kanagawa 252-0374 Japan; 5 School of Pharmacy, Kitasato University, 5-9-1 Shirokane, Minato-Ku, Tokyo, 108-8641 Japan

**Keywords:** Polypharmacy, Prescription drug monitoring programme, *Pro re nata*, Psychotropic drugs

## Abstract

**Background:**

*Pro re nata* (PRN) medication is used “as needed” for symptoms such as agitation and insomnia, in addition to regular daily pharmacotherapy of mental disorders. However, there is no high-quality evidence on the effectiveness of psychotropic PRN medications and concerns have been raised about their potential to contribute to polypharmacy. This study introduced a psychotropic PRN prescription-monitoring programme for psychiatric inpatients with the aim of examining the change before and after the implementation of the programme.

**Method:**

This study included 389 patients admitted to the psychiatric department between 1 July 2021 and 30 June 2023. The psychotropic PRN prescription-monitoring programme was implemented in July 2022, and the participants were classified into monitoring and non-monitoring groups. Demographic data (age, sex, and diagnosis), regular prescriptions before admission and at discharge, psychotropic PRN prescriptions before admission and at discharge, and the total number of psychotropic PRN prescriptions during hospitalisation were compared between the two groups. Data on psychotropic prescription were collected by psychotropic category. The significance level of 5% was set at 1.67 × 10^−3^ using the Bonferroni correction for multiple testing.

**Results:**

The psychotropic PRN prescription *ratio* at discharge in the monitoring group was 9.3%, which was significantly lower than the 28.1% in the non-monitoring group. The percentage of patients with a PRN prescription during hospitalisation in the monitoring group was 29.8%, which was significantly lower than the 64.5% in the non-monitoring group. In the non-monitoring group, there was no statistically significant difference in the number of psychotropic drugs prescribed regularly before and after admission. However, in the monitoring group, the number of psychotropic drugs in the regular prescriptions at discharge was 1.87 ± 1.24, which was significantly lower than 2.47 ± 1.90 in the regular prescription before admission.

**Conclusions:**

Our findings suggest that a psychotropic PRN prescription-monitoring programme may contribute to the elimination of polypharmacy, including regular prescriptions. Further research is required to optimise psychotropic PRN prescriptions and reduce polypharmacy.

## Background

Pharmacotherapy plays an important role in treating mental disorders. Several guidelines recommend monotherapy as a first-line strategy for some mental disorders [1–6]. Polypharmacy may be necessary for some conditions, such as treatment resistance, but psychotropic polypharmacy is common in clinical practice [7–9] despite limited evidence of superior efficacy and a high risk of adverse events [10]. Reducing polypharmacy may reduce iatrogenesis and improve adherence to medication. Therefore, reducing polypharmacy is a critical issue in psychiatry.

*Pro re nata* (PRN) prescribing has been noted as one of the factors associated with psychotropic polypharmacy [11–13]. PRN psychotropic medications are used “as needed” for symptoms such as agitation and insomnia, in addition to regular daily pharmacotherapy. PRN psychotropic medications are prescribed for 70–90% of patients hospitalised as a result of psychiatric disorders [14, 15]. Another study in a single-centre involving 205 patients with schizophrenia reported that the use of PRN psychotropic drugs was 0–4.18 times/day, with a mean of 0.48 times/day [11]. These reports suggest that psychotropic PRN is frequently used.

Psychotropic PRN medication is a widely used treatment; however, there is no high-quality evidence supporting the effectiveness of psychotropic PRN medications and their use is based on clinical experience and habits [16]. Guidelines for schizophrenia, bipolar disorder, major depressive disorder, insomnia and anxiety disorders [1–6, 17–19] provide little guidance or are vague on the use of PRN prescriptions to manage symptoms such as insomnia and anxiety. The National Institute for Health and Care Excellence guidelines on violence and aggression [20] recommend against automatically prescribing PRN drugs on admission. Furthermore, they suggested that PRN drug use should be re-examined weekly. If PRN drugs are not used after the previous re-examination, prescription discontinuation should be considered. Despite these recommendations, psychotropic PRN prescription use has not been adequately evaluated in psychiatric inpatients [11], and a system for monitoring psychotropic PRN prescriptions is needed [11, 21]. Previous studies have reported that pharmacovigilance programmes by pharmacologists/pharmacists contribute to the detection and resolution of medication problems in patients with psychiatric disorders [22]. In addition, collaboration between clinical pharmacists and psychiatrists has also been reported to reduce polypharmacy, including PRN medication use [23–26]. Therefore, multidisciplinary prescription monitoring, including pharmacologists/pharmacists, may contribute to reducing inappropriate PRN prescriptions.

Regarding the relationship between psychotropic PRN prescriptions and regular prescriptions, a higher *ratio* of monotherapy and the absence of other psychotropics in regular prescriptions may reduce the use of PRN psychotropic medications [27]. Based on the results of previous studies, efforts to reduce psychotropic PRN prescriptions may reduce psychotropic polypharmacy. Therefore, we developed a psychotropic PRN prescription-monitoring programme for psychiatric inpatients to reduce and optimise psychotropic PRN prescriptions. This study aimed to examine the change before and after the implementation of a psychotropic PRN prescription-monitoring programme on inpatients with psychiatric disorders.

## Methods

### PRN prescription-monitoring programme

**Fig. 1:**
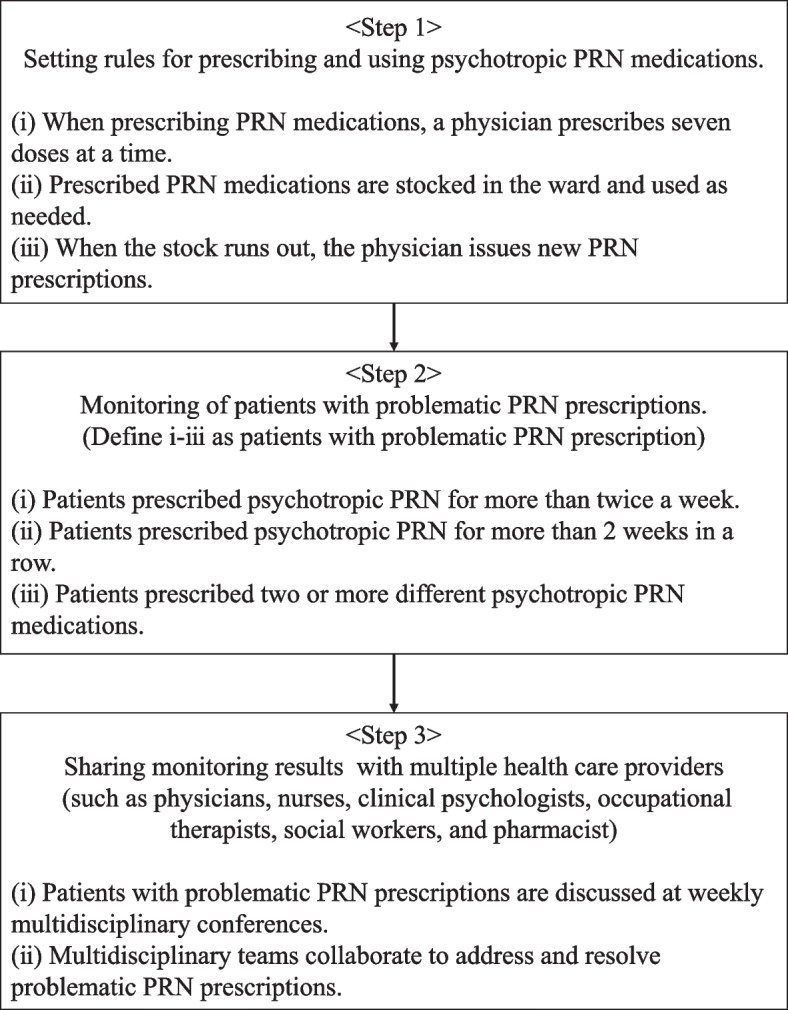
PRN monitoring programme overview

The PRN prescription-monitoring programme for inpatients with psychiatric disorders was initiated at the Department of Psychiatry, Kitasato University Hospital, on 5 July 2022. The programme (Fig. 1) was conducted weekly, as part of a multidisciplinary conference involving physicians, nurses, clinical psychologists, occupational therapists, social workers, and pharmacists, and confirmed and discussed treatment plans, treatment status, and post-discharge support for all inpatients. The PRN monitoring programme was carried out during about 10 min of this conference. The pharmacist checked the PRN prescription status of all inpatients and identified patients with problematic PRN prescriptions. The definition of patients with problematic PRN prescription in this programme is shown in step 1 and 2 of Fig. 1. The monitoring results were shared with the attendees at the multidisciplinary conference and solutions for problematic PRN prescriptions were discussed, including consideration of the purpose of PRN prescriptions, the duration of PRN prescriptions, and methods of reducing PRN prescriptions. The attending physician was then advised by the pharmacist and experienced psychiatrists to consider discontinuing PRN prescriptions that were without any clear purpose. In addition, the pharmacist and experienced psychiatrists suggested adjusting regular prescriptions as a way of reducing PRN prescriptions, while the nurse, occupational therapist, and clinical psychologist suggested alternative methods such as psychological intervention. After these discussions, the status of PRN prescriptions was checked again at the next week's conference.

### Participants

This study aimed to examine changes in pharmacotherapy before and after implementing the PRN prescription-monitoring programme. Therefore, all patients admitted to the Department of Psychiatry at Kitasato University Hospital between 1 July 2021 and 30 June 2023 were included. The programme was introduced in July 2022, so patients admitted between July 2021 and June 2022 were not part of the monitoring programme. Patients were informed about the purpose and procedures of the study and were given the option to opt-out. Prescription monitoring was conducted once a week; therefore, patients who were hospitalised for less than six days were excluded. If the same patient was admitted to hospital more than once during the study period, each admission was counted as a separate subject. This study included 389 subjects, covering all age groups.

This study was approved by the Ethics Committee of Kitasato University Hospital (approval no. B23-074, date of approval: 27 September 2023) and conducted in accordance with the Declaration of Helsinki.

### Data collection

We investigated patients’ demographic data (age, sex, and diagnosis), regular prescriptions before admission and at discharge, psychotropic PRN prescriptions before admission and at discharge, and the total number of psychotropic PRN prescriptions during hospitalisation. These data were extracted from the patient’s medical record. Diagnoses were classified according to the Diagnostic and Statistical Manual of Mental Disorders, 5th edition (DSM-5) [28]. Data on psychotropic prescription were collected according to psychotropic category. Psychotropic drugs were categorised as antipsychotics, anxiolytics and hypnotics, antidepressants, mood stabilisers and antiepileptics, and anticholinergic drugs.

### Statistical analysis

Patients were divided into two groups: the non-monitoring group consisting of those admitted on or before 30 June 2022 and the monitoring group consisting of those admitted on or after 1 July 2022. Sex (female *ratio*) and age (mean, SD) were calculated for each group. The proportion of diagnoses based on the DSM-5 was calculated for each group. Additionally, we performed a Mann–Whitney U-test to compare the number of psychotropic drugs in regular prescriptions at discharge between the two groups by drug category.

The psychotropic PRN prescription *ratios* before admission and at discharge were compared between the two groups using the chi-square test. The psychotropic PRN prescription *ratio* represents the proportion of patients who had been given a PRN prescription, either before or after admission. The psychotropic PRN prescription *ratio* at discharge was also compared according to the drug category. In addition, the percentage of patients with psychotropic PRN prescriptions during hospitalisation was compared between the two groups using the chi-square test. The total number of psychotropic PRN prescriptions during hospitalisation was divided by the number of hospital days to calculate the average number of psychotropic PRN per day. This number was compared between the two groups by drug category using the Mann–Whitney U-test.

Finally, in both monitoring and non-monitoring groups, the number of psychotropic drugs prescribed regularly before admission was compared with the number at discharge for each drug category using the Wilcoxon signed-rank sum test.

The significance level of 5% was set at 1.67 × 10 ^−3^ (0.05/30) using the Bonferroni correction for multiple testing across 32 tests. All statistical analyses were performed using the IBM SPSS Statistics 26.0 (IBM Corp., Armonk, NY, USA).

## Results

Table 1 shows the sex, age, and the DSM-5-based diagnoses of the patients. In both groups, the female ratio was approximately 60%, and the average age was 53 years old. Schizophrenia spectrum and other psychotic disorders accounted for about half of the patients in both the monitoring and non-monitoring groups. In both groups, bipolar disorder and related disorders accounted for 10–15% of the patients, and depressive disorders accounted for 15–20% of the patients. All other diagnoses were less common, accounting for less than 10% of the total diagnoses.

**Table 1:** Sex, age, and diagnosis of all patients according to the Diagnostic and Statistical Manual of Mental Disorders 5th edition

	Monitoring group (*n* = 161)	Non-monitoring group (*n* = 228)	All (*n* = 389)
Sex (female *ratio*, %)	102 (63.4%)	130 (57.0%)	232 (59.6%)
Age (mean, SD)	53.31 ± 18.12	53.21 ± 19.07	53.25 ± 18.66
Diagnosis(including co-morbid diagnoses)			
Schizophrenia spectrum and other psychotic disorders	78 (48.4%)	130 (57.0%)	208 (53.5%)
Depressive disorders	30 (18.6%)	34 (14.9%)	64 (16.5%)
Bipolar and related disorders	25 (15.5%)	23 (10.1%)	48 (12.3%)
Neurodevelopmental disorders	10 (6.2%)	15 (6.6%)	25 (6.4%)
Substance-related and addictive disorders	6 (3.7%)	15 (6.6%)	21 (5.4%)
Neurocognitive disorders	5 (3.1%)	10 (4.4%)	15 (3.9%)
Personality disorders	2 (1.2%)	7 (3.1%)	9 (2.3%)
Dissociative disorders	2 (1.2%)	6 (2.6%)	8 (2.1%)
Feeding and eating disorders	4 (2.5%)	4 (1.8%)	8 (2.1%)
Somatic symptoms and related disorders	1 (0.6%)	3 (1.3%)	4 (1.0%)
Anxiety disorders	0 (0%)	2 (0.9%)	2 (0.5%)
Obsessive–compulsive and related disorders	1 (0.6%)	1 (0.4%)	2 (0.5%)
Trauma and stressor-related disorders	0 (0%)	2 (0.9%)	2 (0.5%)

Table 2 shows regular prescriptions of the patients at discharge. Although the number of psychotropic drugs is lower in the monitoring group than in the non-monitoring group, there were no statistically significant differences in the number of psychotropic drugs between the two groups.

**Table 2:** Comparison between the monitoring and non-monitoring groups regarding the number of psychotropic drugs at discharge

Regular prescription at discharge	Monitoring group (*n* = 161)	Non-monitoring group (*n* = 228)	*p*-value
Number of all psychotropic drugs	1.87 ± 1.24	2.27 ± 1.65	0.03
Number of antipsychotics	0.86 ± 0.65	1.05 ± 0.77	0.02
Number of anxiolytics and hypnotics	0.47 ± 0.72	0.58 ± 1.04	0.81
Number of antidepressants	0.21 ± 0.41	0.25 ± 0.52	0.78
Number of mood stabilizers and antiepileptics	0.25 ± 0.49	0.23 ± 0.45	0.87
Number of anticholinergics	0.07 ± 0.26	0.15 ± 0.38	0.04

As the level of significance, *p* < 1.67 × 10^–3^, was within the 5% significance level based on the Bonferroni correction, it was considered in the multiplicity of the tests

Table 3 shows the psychotropic PRN prescriptions for patients. There was no significant difference in the psychotropic PRN prescription *ratio* before admission between the two groups. The psychotropic PRN prescription *ratio* at discharge in the monitoring group was 9.3%, which was significantly lower than 28.1% in the non-monitoring group (*p* = 5.9 × 10^–6^). In the non-monitoring group, the psychotropic PRN prescription *ratio* before admission was 25.4%, and at discharge it was 28.1%, higher than before admission. The antipsychotic PRN prescription *ratio* at discharge was significantly lower in the monitoring group (5.6%) than in the non-monitoring group (*p* = 1.4 × 10^–5^). The proportion of patients with a PRN prescription during hospitalisation in the monitoring group was 29.8%, which was significantly lower than 64.5% in the non-monitoring group (*p* = 1.7 × 10^–11^). Furthermore, the number of psychotropic PRN prescriptions per hospitalisation day in the monitoring group was 0.29 ± 0.76, significantly lower than 1.33 ± 1.50 in the non-monitoring group (*p* = 3.0 × 10^–16^). By drug category, the number of antipsychotic PRN prescriptions per hospitalisation day in the monitoring group was 0.17 ± 0.60, significantly lower than 0.88 ± 1.10 in the non-monitoring group (*p* = 8.2 × 10^–14^). The number of anxiolytic and hypnotic PRN prescriptions per hospitalisation day in the monitoring group was 0.06 ± 0.27, significantly lower than 0.35 ± 0.80 in the non-monitoring group (*p* = 1.8 × 10^–4^).

**Table 3:** Comparison between the monitoring and non-monitoring groups regarding the psychotropic PRN prescription

	Monitoring group (n = 161)	Non-monitoring group (n = 228)	*p*-value
Psychotropic PRN prescription *ratio* before admission	32 (19.9%)	58 (25.4%)	0.20
Psychotropic PRN prescription *ratio* at discharge	15 (9.3%)	64 (28.1%)	*5.9 × 10^–6^
Antipsychotic PRN prescription *ratio* at discharge	9 (5.6%)	49 (21.5%)	*1.4 × 10^–5^
Anxiolytic and hypnotic PRN prescription *ratio* at discharge	6 (3.7%)	22 (9.6%)	0.03
Antidepressant PRN prescription *ratio* at discharge	1 (0.4%)	0 (0%)	0.40
Anticholinergic PRN prescription *ratio* at discharge	1 (0.6%)	9 (3.9%)	0.04
Patients with a psychotropic PRN prescription during hospitalisation	48 (29.8%)	147 (64.5%)	*1.7 × 10^–11^
Number of psychotropic PRN prescriptions per hospitalisation day	0.29 ± 0.76	1.33 ± 1.50	*3.0 × 10^–16^
Number of antipsychotic PRN prescriptions per hospitalisation day	0.17 ± 0.60	0.88 ± 1.10	*8.2 × 10^–14^
Number of anxiolytic and hypnotic PRN prescriptions per hospitalisation day	0.06 ± 0.27	0.35 ± 0.80	*1.8 × 10^–4^
Number of antidepressant PRN prescriptions per hospitalisation day	0.002 ± 0.02	0.02 ± 0.16	0.22
Number of anticholinergic PRN prescriptions per hospitalisation day	0.05 ± 0.22	0.08 ± 0.31	0.15

As the level of significance, *p* < 1.67 × 10^–3^, was within the 5% significance level based on the Bonferroni correction, it was considered in the multiplicity of the tests

*p < 0.05, after the Bonferroni correction

Tables 4 and 5 compare the number of psychotropic drugs in patients' regular prescriptions before and after hospitalisation. In the non-monitoring group, there was no statistically significant difference in the number of psychotropic drugs administered before and after admission. However, the number of anxiolytics and hypnotics at discharge was 0.58 ± 1.04, which was significantly lower than 0.83 ± 1.09 before admission (*p* = 4.6 × 10^–5^). In the monitoring group, the total number of psychotropic drugs at discharge was 1.87 ± 1.24, which was significantly lower than 2.47 ± 1.90 before admission (*p* = 9.5 × 10^–6^). For each drug category, the number of anxiolytics and hypnotics in the regular prescription at discharge was 0.47 ± 0.72, which was significantly lower than 0.83 ± 1.02 in the regular prescription before admission (*p* = 5.7 × 10^–6^).

**Table 4:** Comparison of regular prescriptions before and after hospitalisation in the non-monitoring group

Non-monitoring group (*n* = 228)	Regular prescription before admission	Regular prescription at discharge	*p*-value
Number of all psychotropic drugs	2.46 ± 1.99	2.27 ± 1.65	0.06
Number of antipsychotics	0.93 ± 0.91	1.05 ± 0.77	0.01
Number of anxiolytics and hypnotics	0.83 ± 1.09	0.58 ± 1.04	*4.6 × 10^–5^
Number of antidepressants	0.28 ± 0.53	0.25 ± 0.52	0.35
Number of mood stabilizers and antiepileptics	0.23 ± 0.49	0.23 ± 0.45	0.99
Number of anticholinergics	0.19 ± 0.45	0.15 ± 0.38	0.13

As the level of significance, *p* < 1.67 × 10^–3^, was within the 5% significance level based on the Bonferroni correction, it was considered in the multiplicity of the tests

^*^p < 0.05, after the Bonferroni correction

**Table 5:** Comparison of regular prescriptions before and after hospitalisation in the monitoring group

Monitoring group (*n* = 161)	Regular prescription before admission	Regular prescription at discharge	*p*-value
Number of all psychotropic drugs	2.47 ± 1.90	1.87 ± 1.24	*9.5 × 10^–6^
Number of antipsychotics	0.89 ± 0.77	0.86 ± 0.65	0.57
Number of anxiolytics and hypnotics	0.83 ± 1.02	0.47 ± 0.72	*5.7 × 10^–6^
Number of antidepressants	0.35 ± 0.65	0.21 ± 0.41	1.8 × 10^–3^
Number of mood stabilizers and antiepileptics	0.30 ± 0.57	0.25 ± 0.49	0.19
Number of anticholinergics	0.11 ± 0.31	0.07 ± 0.26	0.23

As the level of significance, *p* < 1.67 × 10^–3^, was within the 5% significance level based on the Bonferroni correction, it was considered in the multiplicity of the tests

^*^p < 0.05, after the Bonferroni correction

## Discussion

This is the first study to examine the change before and after the implementation of a psychotropic PRN prescription-monitoring programme on inpatients with psychiatric disorders. Psychotropic PRN prescriptions during hospitalisation were significantly less frequent in the monitoring group than in the non-monitoring group, and the number of psychotropic PRN prescriptions per hospitalisation day was significantly lower. Furthermore, the psychotropic PRN prescription rate at discharge was significantly lower in the monitoring group than in the non-monitoring group. These results suggest that the psychotropic PRN prescription-monitoring programme reduced psychotropic PRN prescriptions in inpatients with psychiatric disorders.

PRN prescriptions are influenced by factors such as individual staff, ward and organisational practices, staffing, and environmental conditions [29]. PRN medications are often prescribed early during hospitalisation [14] and may be administered automatically without considering patient preference or usage [11]. Physicians and nurses differ in their use of PRN prescriptions [30]. Healthcare professionals often consider PRN prescription as the first option for patients with insomnia or agitation [21, 30, 31]. However, patients may not always understand why PRN prescriptions are prescribed and can sometimes feel coerced [32, 33]. Many factors are involved in PRN medication prescription, but it is unclear whether patients and prescribing physicians can cooperate in decision-making [34]. The automatic prescription of unnecessary PRN medications may lead to iatrogenesis, including polypharmacy. Since psychotropic PRN prescriptions have been reported to be associated with polypharmacy [11–13], reducing PRN prescriptions may reduce polypharmacy. Some studies have shown that reducing PRN drugs and replacing them with psychological interventions can reduce the patients’ violence and aggression, as well as the need for movement or activity restrictions [35–37]. Reducing PRN medication prescription and improving care, including psychological interventions, may contribute to patient recovery. Reducing PRN prescriptions requires cooperation from medical teams [21]. Monitoring and reviewing PRN prescriptions with multiple healthcare professionals promote communication among healthcare professionals and patients. As a result, the PRN prescription-monitoring programme can help eliminate automatic and unnecessary PRN prescriptions.

In this study, the total number of psychotropic drugs in regular prescriptions did not significantly change before or after admission in the non-monitoring group. In contrast, the number of psychotropic drugs in the monitoring group was significantly lower at discharge than before admission. PRN medications increase the complexity of medication regimens [38, 39]. Frequent administration of PRN medications can mask the signs and symptoms of underlying diseases [38, 40]. In this study, reducing psychotropic PRN prescriptions may have facilitated treatment with regular prescriptions and optimising regular prescriptions minimised the number of drugs used in treatment by focusing on drug dose adjustment.

By drug category, the number of anxiolytics and hypnotics decreased significantly before and after admission in both groups. In Japan, medical service fees have been revised to reduce irrational psychotropic polypharmacy [41], and a study using a large Japanese administrative claims database reported a decrease in anxiolytics and hypnotics since the 2018 revision [42]. The significant decrease in the number of anxiolytics and hypnotics observed in this study, regardless of monitoring, may be related to recent changes in prescription trends. Additionally, there were no significant differences in the number of antipsychotics, antidepressants, mood stabilisers, antiepileptics, and anticholinergics administered between the monitoring and non-monitoring groups before and after hospitalisation. Therefore, it was not possible to determine from this study which drug categories were reduced by the psychotropic PRN prescription-monitoring programme. Further research is needed in this regard.

## Limitations

This study had some limitations. First, this study investigated inpatients with psychiatric disorders with various diagnoses without assessing outcomes by specific diagnoses, and differences in the frequency of diagnoses between the two groups may have affected the results. Second, the severity of psychiatric symptoms was not assessed using a rating scale. Therefore, it was not possible to determine the impact of the psychotropic PRN monitoring programme on patients' symptoms and social functioning. In addition, this study did not assess what changes in possible outcome parameters such as adverse drug reactions were made by the PRN monitoring programme. Therefore, the extent to which the PRN monitoring programme leads to actual improvements in drug treatment safety and care has not been assessed. Third, we did not evaluate the number of psychotropic PRN prescriptions used per dose and the total number of PRN medications actually taken by patients. Fourth, this was a retrospective analysis conducted at a single facility in Japan and the sample size was small, which may limit the generalisability of the results. Fifth, the monitoring group and the non-monitoring-group had different admission times and, therefore, each group had different healthcare professionals involved in their care. Furthermore, it is possible that the non-monitoring group, whose hospitalisation period was from July 2021 to June 2022, was more affected by the Covid-19 pandemic in terms of anxiety and other psychiatric symptoms. Future comparative studies using parallel groups are required. Further research is needed to optimise psychotropic PRN prescriptions and reduce polypharmacy.

## Conclusion

Our study indicates that a psychotropic PRN prescription-monitoring programme may contribute to the elimination of polypharmacy, including regular prescriptions. This suggests that such programmes can minimize polypharmacy, thereby improving medication management and safety. As PRN medication prescribing is associated with polypharmacy, reducing PRN prescribing may lead to a reduction in polypharmacy. Further research is required to optimise psychotropic PRN prescriptions and reduce polypharmacy. Prospective comparative studies with parallel groups should be conducted with increased sample sizes.

## Data Availability

The datasets generated and/or analysed during the current study are not publicly available owing to ethical reasons but are available from the corresponding author upon reasonable request.

## Abbreviations

DSM-5: The Diagnostic and Statistical Manual of Mental Disorders, 5th edition
PRN: *Pro re nata*
SD: Standard deviation
